# Node of Ranvier remodeling in chronic psychosocial stress and anxiety

**DOI:** 10.1038/s41386-023-01568-6

**Published:** 2023-03-22

**Authors:** Maija-Kreetta Koskinen, Mikaela Laine, Ali Abdollahzadeh, Adrien Gigliotta, Giulia Mazzini, Sarah Journée, Varpu Alenius, Kalevi Trontti, Jussi Tohka, Petri Hyytiä, Alejandra Sierra, Iiris Hovatta

**Affiliations:** 1 SleepWell Research Program, Faculty of Medicine, University of Helsinki, Helsinki, Finland; 2 Department of Psychology and Logopedics, Faculty of Medicine, University of Helsinki, Helsinki, Finland; 3 Neuroscience Center, Helsinki Institute of Life Science HiLIFE, University of Helsinki, Helsinki, Finland; 4 A.I. Virtanen Institute for Molecular Sciences, University of Eastern Finland, Kuopio, Finland; 5 Department of Pharmacology, Faculty of Medicine, University of Helsinki, Helsinki, Finland

**Keywords:** Neuroscience, Molecular biology, Stress and resilience, Cellular neuroscience

## Abstract

Differential expression of myelin-related genes and changes in myelin thickness have been demonstrated in mice after chronic psychosocial stress, a risk factor for anxiety disorders. To determine whether and how stress affects structural remodeling of nodes of Ranvier, another form of myelin plasticity, we developed a 3D reconstruction analysis of node morphology in C57BL/6NCrl and DBA/2NCrl mice. We identified strain-dependent effects of chronic social defeat stress on node morphology in the medial prefrontal cortex (mPFC) gray matter, including shortening of paranodes in C57BL/6NCrl stress-resilient and shortening of node gaps in DBA/2NCrl stress-susceptible mice compared to controls. Neuronal activity has been associated with changes in myelin thickness. To investigate whether neuronal activation is a mechanism influencing also node of Ranvier morphology, we used DREADDs to repeatedly activate the ventral hippocampus-to-mPFC pathway. We found reduced anxiety-like behavior and shortened paranodes specifically in stimulated, but not in the nearby non-stimulated axons. Altogether, our data demonstrate (1) nodal remodeling of the mPFC gray matter axons after chronic stress and (2) axon-specific regulation of paranodes in response to repeated neuronal activity in an anxiety-associated pathway. Nodal remodeling may thus contribute to aberrant circuit function associated with anxiety disorders.

## Introduction

Anxiety disorders are the most common psychiatric disorders, affecting up to 14% of the population [1], with a large socioeconomic burden [2]. Anxiety disorders are complex diseases with both genetic and environmental risk factors [3, 4], however, little is known about the mechanisms underlying these gene-environment interactions. Chronic psychosocial stress increases the risk to develop anxiety disorders [5, 6]. Understanding the mechanisms mediating vulnerability to stress, and resilience to it, is crucial for the development of much-needed treatment and prevention strategies.

By employing the chronic social defeat stress (CSDS) model, a well-validated animal model of psychosocial stress and anxiety-like behavior in male mice [7, 8], we recently demonstrated using four inbred mouse strains that behavioral responses to stress are strongly influenced by genetic background [9]. CSDS, consisting of confrontations between intruder and resident mice, leads to social avoidance in a subset of mice (stress-susceptible), while others retain social approach (stress-resilient) like non-stressed mice. For example, most (~95%) DBA/2NCrl (D2) mice show social avoidance after CSDS, and are thus classified as stress-susceptible, while the majority (~70%) of C57BL/6NCrl (B6) mice are stress-resilient [9].

Our prior unbiased gene expression profiling study, aimed to identify biological pathways mediating stress-induced anxiety and resilience to it, discovered statistical over-representation of myelin-related genes among differentially expressed genes in mice after CSDS [9]. Both the genetic background, as well as resilience and susceptibility to stress modulated this effect. Changes in gene expression were accompanied by strain- and susceptibility/resilience-dependent differences in myelin sheath thickness in the medial prefrontal cortex (mPFC), ventral hippocampus (vHPC), and bed nucleus of stria terminalis (BNST), regions involved in the regulation of anxiety [10]. Altogether, these findings, also supported by others [11–15], highlight the involvement of myelin plasticity in stress response and suggest an important role for myelination in mediating susceptibility and resilience to stress.

Myelin plasticity, or adaptive myelination, encompasses various alterations in myelin structure, density, and function in response to specific experiences and consequent changes in neuronal activity [16, 17]. In addition to changes in myelin thickness and de novo myelination of previously non-myelinated axons or axonal segments, myelin plasticity involves modulation of nodes of Ranvier [18], small unmyelinated segments between myelin sheaths which enable saltatory conduction [19]. They consist of unmyelinated node gaps containing a high density of voltage-gated sodium ion channels that are flanked by paranodes, where myelin sheaths attach to axons. Juxtaparanodes are located adjacent to paranodes, and contain a high density of potassium channels [19–21]. Similar to changes in myelin thickness and sheath length node of Ranvier remodeling can also have profound changes on axonal conduction [22–25], and thus critically impact connectivity of neuronal networks [26]. Whether node of Ranvier morphology in the cortical gray matter is affected in chronic psychosocial stress, and whether activity-dependent nodal changes occur in anxiety-associated circuits remain unclear.

Here, we first investigated whether modulation of node of Ranvier structure occurs in response to CSDS, and whether these changes are influenced by genetic background or resilience or susceptibility to stress-induced social avoidance, a symptom of anxiety. We identify strain-dependent effects of chronic stress on nodal structures using a novel 3D segmentation method, allowing refined structural node analysis. In a second set of experiments, we test neuronal activation as a possible mechanism underlying morphological changes of nodes of Ranvier, and demonstrate that repeated chemogenetic activation of the vHPC-to-mPFC pathway reduces anxiety-like behavior and induces axon-specific shortening of paranode length, suggesting that activity and axon-specific nodal remodeling may contribute to shaping of neuronal connectivity in anxiety circuits.

## Materials and methods

Detailed materials and methods can be found in the Supplementary information.

### Animals

C57BL6/NCrl (B6) and DBA/2NCrl (D2) male mice (5 weeks old upon arrival, Charles River Laboratories) were purchased for all experiments. All animal procedures were approved by the Regional State Administration Agency for Southern Finland (ESAVI/2766/04.10.07/2014 and ESAVI/9056/2020) and conducted in accordance with directive 2010/63/EU of the European Parliament and of the Council.

### Experiment 1: CSDS

CSDS was performed as previously described [9, 27]. Seven-week-old male mice underwent a 10-day CSDS paradigm, consisting of a 5–10 min daily physical confrontation and constant sensory interaction. One day after the end of CSDS mice were tested for social avoidance (SA). A social interaction (SI) ratio was calculated for each mouse. Susceptible mice were defined as having SI ratios below a boundary defined as the strain-specific control mean score minus one standard deviation [9].

### RNA-sequencing and differential gene expression analysis

We re-analyzed RNA sequencing data from brain samples of B6 and D2 mice after CSDS, published by us previously [9] (GEO accession GSE109315). Briefly, mice were sacrificed 6–8 days after the last CSDS session and RNA was extracted. Sequencing libraries were prepared with ScriptSeq v2 RNA-seq library preparation kit (Epicentre) and sequencing was performed on NextSeq500 (single-end 96 bp; Illumina). Differential expression analysis on voom normalized [28] gene expression values was performed using limma eBayes [29, 30], comparing resilient and susceptible mice to their same-strain controls. Here, we conducted gene set enrichment analysis (GSEA Desktop v4.1.0 [31, 32]) using the differential expression results published in [9].

### Experiment 2: DREADD—stereotaxic surgery

Stereotaxic surgeries were performed under isoflurane anesthesia. AAV_retro_-Cre was injected into the mPFC (AP: +2.22 mm, ML: ±0.35 mm, DV: −2.1 mm) for retrograde transport to vHPC neurons projecting to the mPFC. For the vHPC viral construct (AP: −3.4 mm, ML: ±2.9 mm, DV: −4.5 mm), mice were randomly assigned to receive either the control virus (AAV8-Dio-mCherry) or the DREADD virus (AAV8-Dio-hM3D(Gq)-mCherry).

### Experiment 2: DREADD—behavioral testing and CNO injections

Mice were tested in the elevated zero maze (EZM) test 20–30 min after receiving a clozapine-N-oxide (CNO, 1 mg/kg) injection (i.p). The total time spent in open and closed areas was recorded over 5 min and analyzed using Ethovision XT10 software. CNO injections were continued once per day for a total of 15 days.

On day 13, prior to receiving CNO, the open field test (OFT) was performed. Each mouse was allowed to explore an arena for 5 min. The time the mice spent in the center vs. periphery was computed. On day 14, the EZM was repeated with slight modifications (EZM2). To test for effects of chronic vHPC-mPFC activation on social avoidance behavior, we performed the SA test on day 15. Each mouse went through two trials of the SA test similarly as after CSDS. To explore whether the chronic activation had affected the acute response to CNO, we performed an additional test of anxiety-like behavior following a priming injection (day 16). Each mouse received an injection of CNO and after 20–30 min they were tested in an elevated plus maze (EPM).

### Nodes of Ranvier immunohistochemistry

Mice were transcardially perfused, post-fixed and brains cut into 35–40 µm sections. The used antibodies were: rabbit anti-Nav1.6 (1:250, #ASC-009, Alomone labs), mouse anti-CASPR (1:5000, #75-001, Neuromab), goat anti-rabbit Alexa Fluor 568 (1:400, #A-11011, Thermo Fisher Scientific), goat anti-mouse Alexa Fluor 488 (1:400, #A28175, Thermo Fisher Scientific), and goat anti-mouse Alexa 647 (1:400, #ab150115, Abcam).

### Imaging

Imaging was performed with ZEISS LSM 880 Confocal Laser Scanning microscope with AiryScan (Zeiss). To image individual nodes within a field of view, a region around a node was cropped, and a z-stack of the cropped region was acquired. Z-stacks were acquired at a resolution of 0.04 × 0.04 × 0.10 µm.

### 3D segmentation and morphometry of paranodes and juxtaparanodes

We developed an automated pipeline to segment and analyze the morphology of paranodes and juxtaparanodes, as well as to measure the length of nodes of Ranvier in the acquired 3D microscopy images (Fig. S4).

### Statistical analysis

We assessed group differences in node and paranode morphology using a mixed model design, in which individual mice and paranode dependency (two paranodes originate from the same node) were treated as random factors and group (control, resilient, and susceptible) and staining batch as fixed factors. Unpaired (two-tailed) Student’s *t* test or a Mann–Whitney *U* test were used to assess behavioral test differences. Two-way repeated ANOVA was used to analyze repeated testing in the EZM task. Mixed model was performed using R (4.2.2) and other statistical analyses using Prism 8.

## Results

### Node of Ranvier-related genes are differentially expressed in response to chronic psychosocial stress

**Fig. 1:**
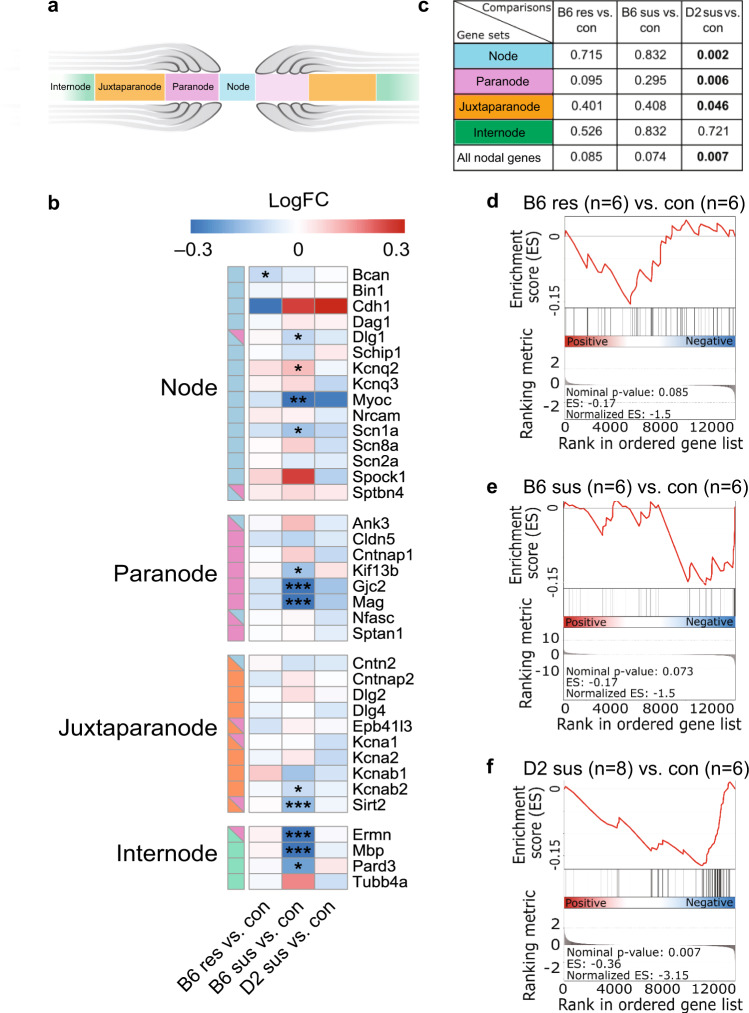
Differential expression of nodes of Ranvier genes after chronic social defeat stress. **a** Schematic representation of the node of Ranvier subregions. **b** Heatmap showing the expression fold change (logFC) and significance of differential expression for genes associated with nodes of Ranvier subcomponents (node, light blue; paranode, pink; juxtaparanode, orange; internode, green) in B6 and D2 defeated mice in the mPFC. **c** Gene Set Enrichment Analysis (GSEA) of genes associated with the individual structural subcomponents of the nodes of Ranvier (node, paranode, juxtaparanode, internode) and combined analysis of all nodal genes (*N* = 40 genes, see Supplementary Table 1). **d**–**f** GSEA enrichment score figures of all nodal genes. The top portion of the plot shows the enrichment score, which reflects the degree of overrepresentation of the gene set at the top or bottom of a ranked gene list. The middle portion of the plot shows the position of the genes in the ranked list. The bottom portion of the plot shows the value of the ranking metric as the analysis walks down the list of ranked genes. B6: C57BL/6NCrl; D2: DBA/2NCrl; Con control, Res resilient, Sus susceptible. *p < 0.05, **p < 0.01, ***p < 0.001.

In our previous study, we discovered changes in the expression of myelin-related genes in response to CSDS [9]. These changes were accompanied by differences in myelin thickness, which occurred in a brain region- and strain-dependent manner, altogether demonstrating myelin plasticity as an integral part of the stress response [9]. These findings prompted us to further investigate whether chronic stress impacts other myelin features, such as nodes of Ranvier. To investigate differential expression of genes involved in the structure and function of nodes of Ranvier (Fig. 1a and Table S1), we re-analyzed our previously published RNA-sequencing dataset from the mPFC (including the prelimbic, infralimbic, and anterior cingulate cortex regions) of B6 and D2 mice, performed 1 week after cessation of a 10-day CSDS [9]. We examined 40 genes associated with the Gene Ontology term node of Ranvier. Twelve genes (30%) were differentially expressed in B6 susceptible mice compared to controls, with 11 of them (28%) being expressed at a lower level in the susceptible compared to control mice (Fig. 1b). These genes encoded for voltage-dependent ion channels (e.g., *Kcnq2*; Kv7.2 and *Scn1a*; Nav1.1) and structural components of the nodes (e.g., *Gjc*; Connexin-47; *Mag;* Myelin associated glycoprotein). In addition, to examine whether genes encoding for structural subcomponents of the nodes of Ranvier (Fig. 1a) were over-represented among the up- or downregulated genes, we conducted Gene Set Enrichment Analysis (GSEA) [31] on differential gene expression lists of B6 resilient, B6 susceptible, and D2 susceptible mice compared to controls (Fig. 1c, d). As D2 resilient mice are found very infrequently, they were not available in the cohort used for this experiment. We found that paranodal, juxtaparanodal, and nodal genes were significantly overrepresented among the downregulated genes in the D2 susceptible mice compared to same-strain controls. In B6 susceptible mice, we observed a statistical trend for overrepresentation of node of Ranvier genes among the downregulated genes. GSEA performed with all 40 genes associated with nodes of Ranvier produced similar results (Fig. 1c, d). Taken together, these results suggest that chronic psychosocial stress alters the expression levels of genes involved in node of Ranvier structure and function in the mPFC of B6 and D2 susceptible mice.

### Strain-dependent alterations of mPFC nodal morphology after chronic psychosocial stress

To investigate whether the observed gene expression differences are associated with changes in node of Ranvier morphology, we developed a novel 3D reconstruction analysis of nodal subdomains. Particularly in gray matter nodes of Ranvier can present highly variable orientations, and as a result, two-dimensional maximum projection analysis of morphology can induce significant distortions of measured parameters. By measuring nodes as they exist in three-dimensional space, we can obtain accurate morphological measures. We analyzed nodal subdomains after CSDS in the anterior cingulate cortex subregion of the mPFC, a critical hub for the regulation of anxiety [33]. This brain region contains several short- and long-range connections [34] as well as excitatory and inhibitory neurons, both of which are known to be myelinated [35]. To investigate whether genetic background or susceptibility or resilience to stress influence nodal modifications in response to stress, we conducted the morphological analysis both in B6 and D2 mice, classified either as stress-resilient or -susceptible based on social avoidance behavior following CSDS [9, 27] (Fig. S1).

**Fig. 2:**
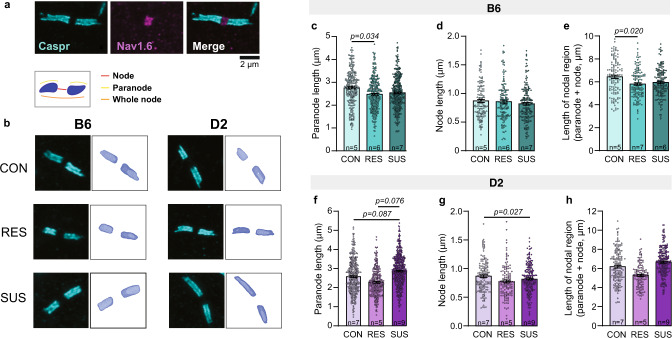
Strain-dependent changes in nodal morphology after chronic social defeat stress. **a**, **b** Examples of 3D reconstructed paranodes in the anterior cingulate cortex layer V/VI in B6 and D2 mice after chronic social defeat stress. Quantification of paranode length (**c**, **f**), node width (**d**, **g**), total nodal region length (**e**, **h**). **c** Paranode length B6: Con = 266 paranodes from 5 mice, Res = 311 paranodes from 6 mice, Sus = 286 paranodes from 7 mice. Statistical differences were identified by linear mixed-effect modeling with pairwise comparisons: Con vs. Res, *t*(13.5) = −2.356, *p* = 0.034; Con vs. Sus, *t*(13.8) = −1.488, *p* = 0.159; Res vs. Sus, *t*(9.4) = 1.148, *p* = 0.279. **d** Node length B6: Con = 132 nodes from 5 mice, Res = 152 nodes from 6 mice, Sus = 168 nodes from 7 mice. Con vs. Res, *t*(9.4) = −0.141, *p* = 0.891; Con vs. Sus, *t*(9.5) = −0.972, *p* = 0.355. **e** Nodal region length B6: Con = 124 nodes from 5 mice, Res = 136 nodes from 6 mice, Sus = 152 nodes from 7 mice. Con vs. Res, *t*(13.6) = −2.630, *p* = 0.020; Con vs. Sus, *t*(13.7) = −1.628, *p* = 0.126. **f** Paranode length D2: Con = 334 paranodes from 7 mice, Res = 274 paranodes from 5 mice, Sus = 476 paranodes from 9 mice, Con vs. Res, *t*(16.6) = 0.040, *p* = 0.968; Con vs. Sus, *t*(16.8) = 1.889, *p* = 0.076; Res vs. Sus, *t*(10.5) = 1.885, *p* = 0.087. **g** Node length D2: Con = 151 nodes from 7 mice, Res = 131 nodes from 5 mice, Sus = 215 nodes from 9 mice. Con vs. Res, *t*(17.5) = −0.487, *p* = 0.632; Con vs. Sus, *t*(18.1) = −2.402, *p* = 0.027. **h** Nodal region length D2: Con = 154 nodes from 7 mice, Res = 124 nodes from 5 mice, Sus = 219 nodes from 9 mice. Con vs. Res, *t*(15.7) = 0.043, *p* = 0.966; Con vs. Sus, *t*(15.6) = 1.035, *p* = 0.317). B6: C57BL/6NCrl; D2: DBA/2NCrl; Con control, Res resilient, Sus Susceptible.

We found that B6 resilient mice had shorter paranodes (by 10.3%), identified by contactin-associated protein (CASPR) immunoreactivity (Fig. 2a, b) compared to same strain controls (Fig. 2c). In D2 mice we found a statistical trend toward increased paranode length in susceptible mice compared both to same strain resilient (by 27.0%) and control mice (by 11.1%) (Fig. 2f). Moreover, node width, defined as the distance between two flanking paranodes (Fig. 2a, b), was shorter (by 5.2%) in D2 susceptible mice compared to same strain control mice (Fig. 2g), but not in B6 mice (Fig. 2d). In accordance with shorter paranodes, total nodal region length was shorter in resilient B6 mice (by 8.4%) compared to same strain controls (Fig. 2e, h). These changes in paranode length are similar in magnitude with previous findings in multiple sclerosis, in which paranodal changes associate with predicted changes in conduction velocity [36].

### Strain-dependent alterations of nodal morphology in forceps minor

In addition to cortical gray matter, we analyzed nodal morphology in response to CSDS in the forceps minor (Fig. S2a), a white matter tract adjacent to the mPFC. Paranode length was longer in B6 stress-resilient (by 7.0%) compared to same strain controls, and there was a statistical trend toward longer paranodes (by 4.4%) in stress-susceptible mice (Fig. S2b). Paranode length in D2 mice or node width in either B6 or D2 mice did not change in response to CSDS (Fig. S2c–e). Altogether, our analysis demonstrates that chronic stress alters node of Ranvier morphology both in the mPFC gray matter and in the forceps minor white matter. These effects were strongly influenced by genetic background and brain region, and they depended on the individual stress response.

### Repeated activation of the vHPC-mPFC pathway reduces anxiety-like behavior and paranode length in axon-specific manner

**Fig. 3:**
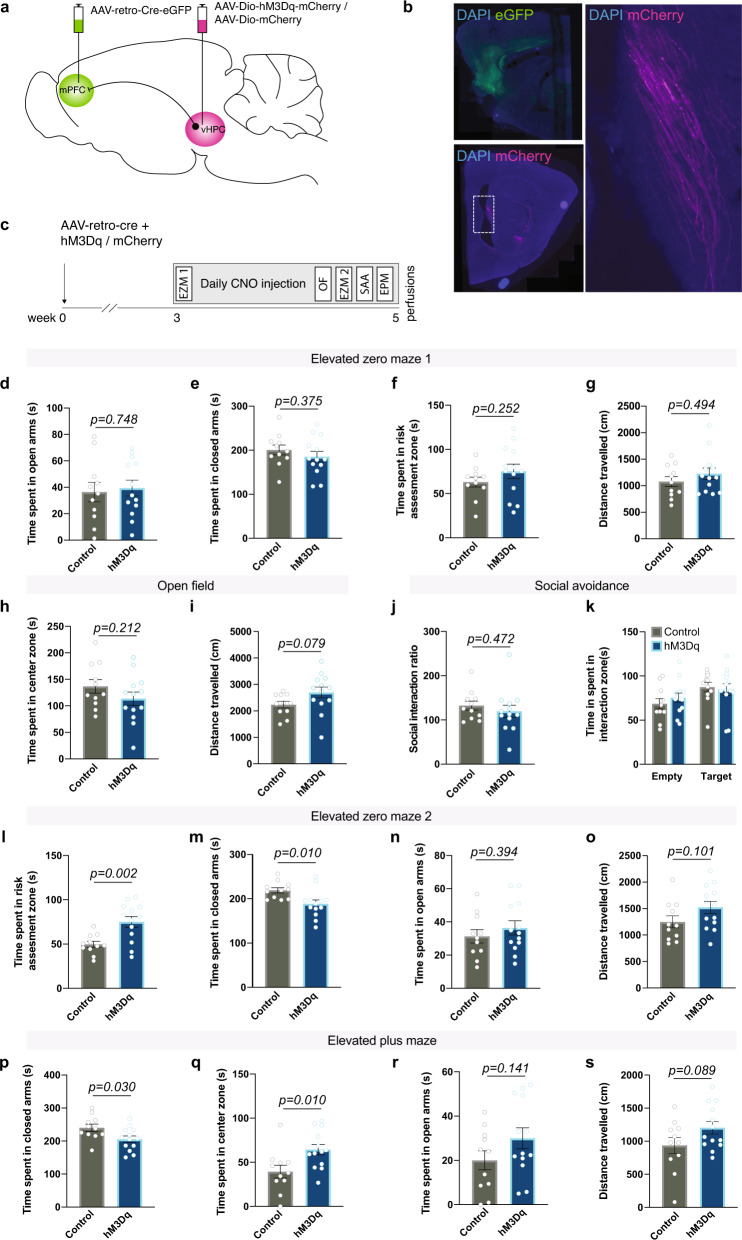
DREADD-mediated activation of vHPC-to-mPFC projection neurons reduces anxiety-like behavior. **a** Projection-specific expression of hM3Dq/mCherry was achieved by injecting a retrograde *Cre*-carrying virus (retro-Cre-eGFP) into the mPFC and a *Cre*-dependent hM3Dq (Dio-hM3Dq-mCherry) or a control virus (Dio-mCherry) into the vHPC CA3 subregion. **b** Representative examples showing eGFP expression in the mPFC and mCherry-expressing axons in the hippocampal fimbria. **c** Experimental timeline. Open zone (**d**), closed zone (**e**) risk assessment (RA) zone (**f**) time and the number of stretch-attend postures in the RA zone (**g**) in the acute elevated zero maze test (EZM1) (two-sided Student’s *t* tests). Time spent in the center of the open field (OF) test (**h**) and distance traveled (**i**) during the OF test (two-sided Student’s *t* tests). **j** Quantification of social interaction ratio in the social approach (SA) task (two-sided Student’s *t* test). **k** Time spent in the interaction zone during empty and target sessions of the SA test (two-way repeated ANOVA: group × session *F*(1,22) = 0.703, *p* = 0.402; session *F*(1,22) = 6.111, *p* = 0.022; group *F*(1,22) = 0.100, *p* = 0.755). Time spent in the open zone (**l**), closed zone (**m**) (two-sided Student’s *t* tests), risk assessment zone (**n**) (two-sided Welch’s *t*-test) and the number of stretch-attend postures in the RA zone (**o**) in the EZM2 test after repeated activation of vHPC-to-mPFC pathway. Time spent in the closed arms (**p**), center zone (**q**), open arms (**r**) and the number of stretch-attend postures (**s**) in the elevated plus maze (EPM) test (two-sided Student’s *t* tests). Control *n* = 11; hM3Dq *n* = 13; Error bars represent ±SEM.

Mechanisms underlying experience-dependent changes in node of Ranvier structure are largely unknown. We hypothesized that neuronal activity may be one such mechanism because it influences oligodendrocyte precursor cell proliferation, oligodendrogenesis, and myelin thickness [37, 38]. Furthermore, transcranial magnetic stimulation affects node of Ranvier width in the stimulated area [23]. However, whether activity-dependent nodal remodeling occurs in circuits involved in the regulation of anxiety-like behavior, and whether such effects are specific to activated axons, are not known. To study the effects of neuronal activation on nodal morphology, we chemogenetically activated neurons projecting from the vHPC to the mPFC, a pathway previously shown to increase anxiety-like behavior upon acute stimulation [39, 40]. To achieve projection-specific expression of excitatory hM3Dq-receptors, we bilaterally injected a retrograde *Cre*-carrying virus (AAV_retro_-Cre-eGFP) into the mPFC, while a *Cre*-dependent hM3Dq (AAV8-Dio-hM3Dq-mCherry) or an mCherry (AAV8-Dio-mCherry) control vector was injected into the vHPC (Fig. 3a) of male C57BL/6NCrl mice. Immunohistochemical analysis confirmed the accuracy of the labeling by showing robust eGFP labeling in the mPFC and mCherry expression in the vHPC CA3/1 regions, as well as eGFP/mCherry-double positive axons along the pathway (Fig. 3b).

Behavioral tests confirmed that the activation of this specific pathway was effective in modulating anxiety-like behavior (Fig. 3c), although the effects differed from those previously published for acute stimulation [39, 40]. We first assessed the effects of an acute chemogenetic activation of Gq signaling on anxiety-like behavior by testing the mice in the elevated zero maze (EZM) 20–30 min after a single clozapine-*N*-oxide (CNO) injection, which activates the hM3Dq receptors. In the EZM, the hM3Dq and control groups spent equal amount of time in the open areas, closed areas, and in the risk assessment zone, and we did not observe differences in stretch-attend postures or general activity (Figs. 3d–g and S3a), suggesting ineffectiveness of the acute manipulation to alter anxiety-like behavior in this assay. After the acute test, we continued daily CNO injections to assess the effects of a repeated manipulation of vHPC-to-mPFC activity on anxiety-like behavior. After 12 days of CNO injections, we carried out the open field (OF) test. Anxiety-like behavior or general activity did not differ between the groups, as demonstrated by similar time spent in the center zone and the distance traveled (Fig. 3h, i). On the following day, and after another CNO injection, we tested the mice in the EZM task. In contrast to the acute CNO effects, after the repeated manipulation hM3Dq mice spent more time in the risk-assessment zone, compared to controls (Fig. 3n). This was accompanied with reduced closed area time (Fig. 3m), with no differences in the time spent in the open areas (Fig. 3l) or general activity (Fig. S3b). The behavior of the control mice did not differ between the acute and chronic EZM tests (Fig. S3c–f), supporting the feasibility of repeated testing in the EZM.

On the following day (after 14 days of CNO injections), we tested the effects of the repeated activation of the vHPC-to-mPFC projection on social behavior in the social approach (SA) task. hM3Dq and control mice spent equal amounts of time interacting with a social target (Fig. 3j, k), suggesting no effects on social avoidance behavior.

Lastly, we tested the priming effects of an acute hM3Dq activation on anxiety-like behavior. After receiving daily CNO injections for 15 days, the mice received a single CNO injection 30 min prior to testing in the elevated plus maze (EPM), to avoid a habituation effect from repeated testing in the EZM. An acute activation in primed animals resulted in reduced exploration of the closed arms in hM3Dq mice, which was accompanied with increased center zone time and increased number of stretch-attend postures compared to controls, with no change in open arm time or in locomotor activity (Figs. 3p–s and S3e). Taken together, our behavioral data demonstrate that prolonged manipulation of vHPC-to-mPFC activity decreases anxiety-like behavior, reflected particularly as increased risk assessment in the EZM test, together with reduced closed arm exploration and increased engagement in stretch-attend postures in the EPM test.

**Fig. 4:**
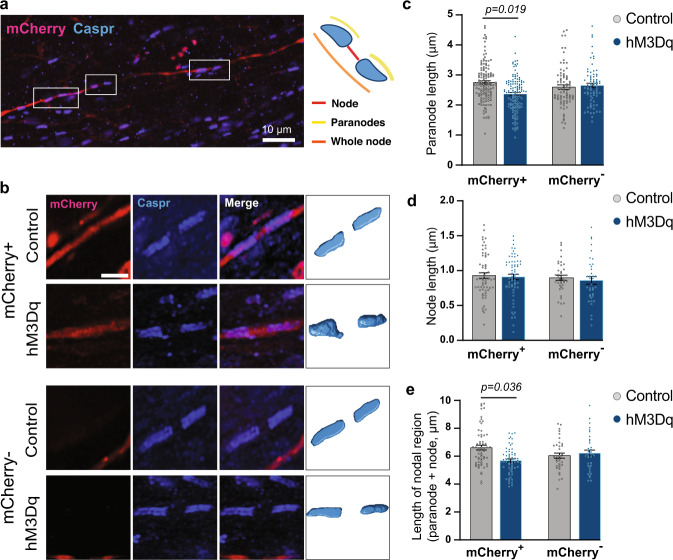
Repeated activation of vHPC-to-mPFC projection neurons reduces paranodal length. **a** A sagittal section containing the vHPC immunolabeled with CASPR antibody in mice expressing an mCherry-labeled hM3Dq or a control virus in vHPC-to-mPFC projection neurons. Outlines demonstrating paranodes along an mCherry-labeled axon. **b** 3D reconstruction of paranodes on labeled and non-labeled axons. **c** Quantification of paranode length (**c** Linear mixed model: Group_virus *t*(16.8) = −0.187, *p* = 0.854; Axon_type *t*(210) = 0.954, *p* = 0.341; Group_virus × Axon_type *t*(205) = −2.463, *p* = 0.015; pairwise comparisons: hM3Dq-mCherry^+^ vs. Control-mCherry^+^ *t*(11) = −2.732, *p* = 0.019; hM3Dq-mCherry^−^ vs. Control-mCherry^−^ *t*(9.7) = −0.047, *p* = 0.963). **d** Quantification of node length (**d** Group_virus *t*(18.6) = −0.060, *p* = 0.953; Axon_type *t*(193) = 0.507, *p* = 0.613; Group_virus × Axon_type *t*(191) = 0.158, *p* = 0.874). **e** Quantification of total nodal region length (**e** Group_virus *t*(17.8) = 0.244, *p* = 0.810; Axon_type *t*(193) = 1.622, *p* = 0.106; Group_virus × Axon_type *t*(191) = −2.569, *p* = 0.011; pairwise comparisons: hM3Dq-mCherry^+^ vs. Control-mCherry^+^ *t*(9.8) = −2.425, *p* = 0.036; hM3Dq-mCherry^−^ vs. Control-mCherry^−^ *t*(9.2) = 0.308, *p* = 0.765). **c** hM3Dq: 73 mCherry− and 123 mCherry+ paranodes from 6 animals; Control: 80 mCherry− and 134 mCherry+ paranodes from 6 animals. **d** hM3Dq: 37 mCherry− and 62 mCherry+ nodes from 6 animals; Control: 39 mCherry− and 61 mCherry+ nodes from 6 animals. **e** hM3Dq: 37 mCherry− and 62 mCherry+ total nodal regions from 6 animals; Control: 37 mCherry− and 64 mCherry+ total nodal regions from 6 animals. **b** Size bar 2 µm. Error bars represent ±SEM.

Finally, we assessed whether DREADD-mediated activation, changing anxiety-like behavior, altered nodal morphology along labeled vHPC-to-mPFC axons. For this, we imaged paranodes (identified with CASPR) on mCherry-expressing (mCherry+) axons within the hippocampal fimbria, where robust labeling of mPFC-projecting vHPC axons was observed in both hM3Dq and control mice (Fig. 4a, b). To tease out manipulation-specific effects, we also studied paranodes on non-labeled axons (mCherry−) within the same brain region. In mice with vHPC-to-mPFC projection neurons expressing hM3Dq, paranodes were significantly shorter (13.9%) than in controls (Fig. 4c). Importantly, the effect was specific for paranodes on mCherry-labeled axons, as no difference in paranode length was present between the groups in non-labeled axons. Furthermore, paranode length did not differ between labeled and non-labeled axons in control mice. In concordance with reduced paranode length, the total nodal region was shorter in hM3Dq-expressing neurons compared to control neurons (Fig. 4e). The DREADD-mediated activation did not affect node width (Fig. 4d). These results demonstrate that manipulation of neuronal activity specifically affected paranode length, and, importantly, the modification occurred in the activated, but not in the non-activated, axons, supporting our hypothesis on activity-dependent remodeling of nodes.

## Discussion

In this study, we first investigated morphology of nodes of Ranvier after chronic psychosocial stress. These studies were based on our initial finding of differential expression of node of Ranvier-related genes within the mPFC of mice exposed to CSDS. The observed gene expression differences were accompanied by altered node of Ranvier morphology. Secondly, to identify mechanisms which could drive nodal remodeling, we assessed whether altered neuronal activity in an anxiety-associated pathway produces nodal changes. In such a pathway, we showed shortened paranodes along DREADD-stimulated axons. Our data demonstrate that DREADD-mediated chronic activation can alter node of Ranvier morphology along active axons, and that such changes can be accompanied by changes in anxiety-like behavior.

We found shorter paranodes in B6 stress-resilient mice compared to controls in the mPFC gray matter after CSDS. In D2 stress-susceptible mice node gap was shorter compared to controls. These findings suggest that nodal remodeling may influence resilience and susceptibility to stress. For example, in B6 the ability to adjust the paranode length, according to the need of the pathway, may promote resilience, a process which may fail in susceptible mice.

Myelination both in the white and gray matter contribute to experience-dependent shaping of neural circuits [18, 41]. Therefore, we also assessed nodal remodeling after chronic stress in the white matter within the forceps minor, the anterior part of the corpus callosum. We found longer paranodes in stress-resilient B6 mice compared to same strain controls, as also previously observed after chronic restraint stress [42]. Different effects of chronic stress on nodal remodeling in the gray and white matter demonstrate that nodal changes are highly brain region-dependent, possibly echoing differences in their activity in response to stress.

What drives the remodeling of the nodes of Ranvier after chronic stress? We hypothesized that behavioral response to chronic stress could be affected by activity-dependent nodal remodeling that takes place selectively in stress-associated circuits. As pathway-specific nodal remodeling in anxiety circuits has to date not been established, we set out to test this hypothesis by using a chemogenetic approach in the vHPC-to-mPFC pathway, previously shown to regulate anxiety-like behavior [39, 40]. We found that prolonged DREADD-mediated activation of vHPC-to-mPFC projection neurons resulted in increased risk-assessment and reduced closed arm exploration, suggesting decreased anxiety-like behavior. An acute inhibition of the pathway has previously been shown to decrease anxiety-like behavior [39, 40], while an acute activation elicited an anxiogenic effect [40]. Altogether, these data demonstrate that vHPC-to-mPFC pathway is critical for the regulation of anxiety-like behavior, yet it underscores a divergent impact of an acute and chronic activation of the pathway on anxiety. We next asked whether nodal changes occur in an activity-dependent and pathway-specific manner in the vHPC-to-mPFC pathway.

Whether activity-related nodal alterations result from generalized activity changes within a brain region, or whether nodal modulation occurs at the level of individual axons, has remained unanswered until recently [43]. Here, we studied whether axon-specific nodal remodeling occurs along axons that mediate anxiety-like behavior. To study this, we used chemogenetics as this method allowed us to repeatedly stimulate specific neurons regulating anxiety-like behavior, while visualizing the manipulated axons for morphological analysis of nodes. Notably, paranodes were specifically modified on activated axons, but not on non-activated axons within the same brain region, suggesting that neuronal activity directly elicits paranodal plasticity along the stimulated axons, as described previously for the formation of myelin sheaths [37, 38], and recently in the motor cortex following learning [43]. Altogether, these findings suggest that node remodeling may play an important role in modulating neuronal communication in anxiety circuits.

Axon-specific modulation of nodes of Ranvier likely adds to fine-tuning of axonal conduction, enabling circuit-specific adaptation to changing processing needs. However, it could also underlie some of the persistent maladaptations associated with stress-related psychiatric disorders. We found that the main nodal effect of CSDS was shortening of paranodes in the mPFC gray matter and lengthening of paranodes in the forceps minor white matter in B6 stress-resilient mice. Similar to the effects observed in the mPFC in B6 stress-resilient mice, DREADD-mediated stimulation led to shorter paranodes in the vHPC-to-mPFC pathway, an effect that was accompanied with reduced anxiety-like behavior. Conversely, in the D2 strain, paranodes appeared longer in the mPFC gray matter in stress-susceptible mice, although these changes did not reach statistical significance. We have previously shown that chronic stress can associate both with thicker or thinner myelin sheaths, depending on whether the mice are stress-susceptible or -resilient and on the genetic background [9]. Similar to dynamic modulation of myelin sheaths [41], bidirectional and brain region-specific modulation of paranodes likely contributes to the capacity to adapt according to the needs of specific circuits and networks [22].

Overall, our data demonstrate genetically controlled remodeling of nodes of Ranvier after chronic stress. Much of the work on myelin plasticity and stress has been conducted using only one mouse strain, typically a B6 substrain. Our findings highlight the need to study more than one genetic background, as appreciating genetically dependent outcomes could improve translational validity of preclinical models. We propose that nodes of Ranvier remodeling associated with CSDS result from stress-induced changes in neuronal activity patterns in distinct gray and white matter regions. Together with other forms of myelin plasticity, i.e., alterations in oligodendrocyte precursor and mature oligodendrocyte function, and changes to myelin distribution and thickness, nodal changes may either promote susceptibility or resilience to stress. We cannot, based on our data, characterize the observed modifications as pathological or adaptive. As anxiety disorders are increasingly considered to involve circuit-level dysfunction, rather than a disruption of a single neurotransmitter system or a single brain region [44], nodal remodeling may contribute to aberrant circuit function in these conditions. Future studies should be aimed not only to understand how nodes of Ranvier remodeling influences conduction velocity along single axons, but to understand its role in circuit- and network level communication. Understanding the mechanisms driving node of Ranvier changes may help pave the way for novel treatment practices for anxiety disorders.

## Supplementary information

Supplementary Table 1.

Supplementary Information

## References

[1] Kessler RC (2007). The global burden of anxiety and mood disorders: putting the European Study of the Epidemiology of Mental Disorders (ESEMeD) findings into perspective. J Clin Psychiatry.

[2] Yang X, Fang Y, Chen H, Zhang T, Yin X, Man J (2021). Global, regional and national burden of anxiety disorders from 1990 to 2019: results from the Global Burden of Disease Study 2019. Epidemiol Psychiatr Sci.

[3] Hettema JM, Neale MC, Kendler KS (2001). A review and meta-analysis of the genetic epidemiology of anxiety disorders. Am J Psychiatry.

[4] Sharma S, Powers A, Bradley B, Ressler KJ (2016). Gene × environment determinants of stress- and anxiety-related disorders. Annu Rev Psychol.

[5] Heim C, Nemeroff CB (1999). The impact of early adverse experiences on brain systems involved in the pathophysiology of anxiety and affective disorders. Biological Psychiatry.

[6] Faravelli C (2012). Childhood stressful events, HPA axis and anxiety disorders. World J Psychiatry..

[7] Krishnan V, Han M-H, Graham DL, Berton O, Renthal W, Russo SJ (2007). Molecular adaptations underlying susceptibility and resistance to social defeat in brain reward regions. Cell..

[8] Hollis F, Kabbaj M (2014). Social defeat as an animal model for depression. ILAR J.

[9] Laine MA, Trontti K, Misiewicz Z, Sokolowska E, Kulesskaya N, Heikkinen A (2018). Genetic control of myelin plasticity after chronic psychosocial stress. Eneuro.

[10] Calhoon GG, Tye KM (2015). Resolving the neural circuits of anxiety. Nat Neurosci.

[11] Zhang H, Yan G, Xu H, Fang Z, Zhang J, Zhang J (2016). The recovery trajectory of adolescent social defeat stress-induced behavioral, 1H-MRS metabolites and myelin changes in Balb/c mice. Sci Rep.

[12] Lehmann ML, Weigel TK, Elkahloun AG, Herkenham M (2017). Chronic social defeat reduces myelination in the mouse medial prefrontal cortex. Sci Rep.

[13] Liu J, Dietz K, Hodes GE, Russo SJ, Casaccia P (2018). Widespread transcriptional alternations in oligodendrocytes in the adult mouse brain following chronic stress: stress alters oligodendrocyte transcription. Devel Neurobio.

[14] Bonnefil V, Dietz K, Amatruda M, Wentling M, Aubry AV, Dupree JL (2019). Region-specific myelin differences define behavioral consequences of chronic social defeat stress in mice. ELife..

[15] Cathomas F, Azzinnari D, Bergamini G, Sigrist H, Buerge M, Hoop V (2019). Oligodendrocyte gene expression is reduced by and influences effects of chronic social stress in mice. Genes Brain Behav.

[16] Mount CW, Monje M (2017). Wrapped to adapt: experience-dependent myelination. Neuron..

[17] Monje M (2018). Myelin plasticity and nervous system function. Annu Rev Neurosci.

[18] de Faria O, Pivonkova H, Varga B, Timmler S, Evans KA, Káradóttir RT (2021). Periods of synchronized myelin changes shape brain function and plasticity. Nat Neurosci.

[19] Lubetzki C, Sol-Foulon N, Desmazières A (2020). Nodes of Ranvier during development and repair in the CNS. Nat Rev Neurol.

[20] Poliak S, Peles E (2003). The local differentiation of myelinated axons at nodes of Ranvier. Nat Rev Neurosci.

[21] Nelson AD, Jenkins PM (2017). Axonal membranes and their domains: assembly and function of the axon initial segment and node of Ranvier. Front Cell Neurosci.

[22] Arancibia-Cárcamo IL, Ford MC, Cossell L, Ishida K, Tohyama K, Attwell D (2017). Node of Ranvier length as a potential regulator of myelinated axon conduction speed. ELife..

[23] Cullen CL, Pepper RE, Clutterbuck MT, Pitman KA, Oorschot V, Auderset L (2021). Periaxonal and nodal plasticities modulate action potential conduction in the adult mouse brain. Cell Rep.

[24] Dutta DJ, Woo DH, Lee PR, Pajevic S, Bukalo O, Huffman WC (2018). Regulation of myelin structure and conduction velocity by perinodal astrocytes. Proc Natl Acad Sci USA.

[25] Ford MC, Alexandrova O, Cossell L, Stange-Marten A, Sinclair J, Kopp-Scheinpflug C (2015). Tuning of Ranvier node and internode properties in myelinated axons to adjust action potential timing. Nat Commun.

[26] Noori R, Park D, Griffiths JD, Bells S, Frankland PW, Mabbott D (2020). Activity-dependent myelination: a glial mechanism of oscillatory self-organization in large-scale brain networks. Proc Natl Acad Sci USA.

[27] Golden SA, Berton O, Russo SJ (2011). A standardized protocol for repeated social defeat stress in mice. Nat Protoc.

[28] Law CW, Chen Y, Shi W, Smyth GK (2014). voom: precision weights unlock linear model analysis tools for RNA-seq read counts. Genome Biol.

[29] Ritchie ME, Phipson B, Wu D, Hu Y, Law CW, Shi W (2015). limma powers differential expression analyses for RNA-sequencing and microarray studies. Nucleic Acids Res.

[30] Phipson B, Lee S, Majewski IJ, Alexander WS, Smyth GK. Robust hyperparameter estimation protects against hypervariable genes and improves power to detect differential expression. Ann Appl Stat. 2016;10:946–63. 10.1214/16-AOAS920 PMC5373812 28367255

[31] Subramanian A, Tamayo P, Mootha VK, Mukherjee S, Ebert BL, Gillette MA (2005). Gene set enrichment analysis: a knowledge-based approach for interpreting genome-wide expression profiles. Proc Natl Acad Sci USA.

[32] Mootha VK, Lindgren CM, Eriksson K-F, Subramanian A, Sihag S, Lehar J (2003). PGC-1α-responsive genes involved in oxidative phosphorylation are coordinately downregulated in human diabetes. Nat Genet.

[33] Bishop S, Duncan J, Brett M, Lawrence AD (2004). Prefrontal cortical function and anxiety: controlling attention to threat-related stimuli. Nat Neurosci.

[34] Riga D, Matos MR, Glas A, Smit AB, Spijker S, Van den Oever MC. Optogenetic dissection of medial prefrontal cortex circuitry. Front Syst Neurosci. 2014;8:00230. 10.3389/fnsys.2014.00230 PMC4260491 25538574

[35] Stedehouder J, Couey JJ, Brizee D, Hosseini B, Slotman JA, Dirven CMF (2017). Fast-spiking parvalbumin interneurons are frequently myelinated in the cerebral cortex of mice and humans. Cereb Cortex.

[36] Gallego-Delgado P, James R, Browne E, Meng J, Umashankar S, Tan L (2020). Neuroinflammation in the normal-appearing white matter (NAWM) of the multiple sclerosis brain causes abnormalities at the nodes of Ranvier. PLoS Biol.

[37] Gibson EM, Purger D, Mount CW, Goldstein AK, Lin GL, Wood LS (2014). Neuronal activity promotes oligodendrogenesis and adaptive myelination in the mammalian brain. Science..

[38] Mitew S, Gobius I, Fenlon LR, McDougall SJ, Hawkes D, Xing YL (2018). Pharmacogenetic stimulation of neuronal activity increases myelination in an axon-specific manner. Nat Commun.

[39] Padilla-Coreano N, Bolkan SS, Pierce GM, Blackman DR, Hardin WD, Garcia-Garcia AL (2016). Direct ventral hippocampal-prefrontal input is required for anxiety-related neural activity and behavior. Neuron..

[40] Parfitt GM, Nguyen R, Bang JY, Aqrabawi AJ, Tran MM, Seo DK (2017). Bidirectional control of anxiety-related behaviors in mice: role of inputs arising from the ventral hippocampus to the lateral septum and medial prefrontal cortex. Neuropsychopharmacology.

[41] Bonetto G, Belin D, Káradóttir RT (2021). Myelin: a gatekeeper of activity-dependent circuit plasticity?. Science..

[42] Miyata S, Taniguchi M, Koyama Y, Shimizu S, Tanaka T, Yasuno F (2016). Association between chronic stress-induced structural abnormalities in Ranvier nodes and reduced oligodendrocyte activity in major depression. Sci Rep.

[43] Bacmeister CM, Huang R, Osso LA, Thornton MA, Conant L, Chavez AR (2022). Motor learning drives dynamic patterns of intermittent myelination on learning-activated axons. Nat Neurosci.

[44] Hare BD, Duman RS (2020). Prefrontal cortex circuits in depression and anxiety: contribution of discrete neuronal populations and target regions. Mol Psychiatry.

