# Supplementary material for: Node of Ranvier remodeling in chronic psychosocial stress and anxiety
Source: Neuropsychopharmacology. 2023 Mar 22;48(10):1532–40. doi: 10.1038/s41386-023-01568-6 (PMC10425340; doi:10.1038/s41386-023-01568-6)
Supplement: Supplementary file 2 — Supplementary Information [file 41386_2023_1568_MOESM2_ESM.pdf]

Supplementary information

Supplementary materials and methods

Animals

C57BL6/NCrl (B6) and DBA/2NCrl (D2) male mice (5 weeks old upon arrival, Charles River Laboratories) were purchased for all experiments and allowed to acclimatize for 10 (Experiment 1: CSDS) or 7 (Experiment 2: DREADD) days before the start of procedures. As aggressor mice in CSDS we acquired Crl:CD1 (CD1, Charles River Laboratories) male mice aged 13-26 weeks. Mice were initially housed in groups (B6 and D2) or individually (CD1) before CSDS at 22 ± 2 °C and humidity 50 ± 15 % on a 12h light/dark cycle (lights on 6:00 – 18:00). After CSDS and stereotactic surgeries all mice were single-housed. They had ad libitum access to water and food except during behavioral experiments. All animal procedures were approved by the Regional State Administration Agency for Southern Finland (ESAVI/2766/04.10.07/2014 and ESAVI/9056/2020) and conducted in accordance with directive 2010/63/EU of the European Parliament and of the Council.

Experiment 1: CSDS

All CSDS behavioral procedures were carried out at the end of the light phase. CD1 mice were first screened for appropriate aggressive behavior during three consecutive days. They had to attack a screener mouse (B6 or D2) on at least two consecutive sessions, within a latency interval of 5-90 s. CSDS was then performed as previously described [1,2]. For CSDS, B6 or D2 mice were introduced into the resident aggressor's compartment for max. 10 min. They were then moved to the other side of the cage, separated from the CD1 by a perforated Plexiglas wall, for the rest of the 24 h. Exposure to CSDS was repeated for 10 days and test mice were subjected to a new aggressor each day. Direct physical contact time was reduced in case of injury. Control mice were housed in pairs in similar cages with no physical confrontation, and cage-mates were changed daily. One day after the end of CSDS, having separated the mice into individual cages, test and control mice were tested for social avoidance (SA). We brought all animals into the testing room 30 min before the test. For the first (no-target) trial, we placed the mouse in the middle of an open

arena with an empty Plexiglas cylinder on one side of the cage. The mouse's movements were tracked with Ethovision XT10 (Noldus Information Technology) for 150 s. The mouse was then returned to the home cage, and the arena cleaned. Immediate after, for the social target trial, the same mouse was placed back into the arena for 150 s, with an unfamiliar CD1 in the cylinder. Time in the interaction zone (IZ) was measured for each trial. A social interaction ratio (time spent in the IZ with the social target present / time spent in the IZ with no target present, multiplied by 100) was calculated for each mouse. For CSDS-exposed animals, susceptible mice were defined as having SI ratios below a boundary defined as the strain-specific control mean score minus one standard deviation[2]. Other CSDS-exposed mice were considered resilient because their social interaction ratio was similar to controls.

RNA-Sequencing and differential gene expression analysis

We re-analyzed RNA sequencing data from brain samples of B6 and D2 mice after CDSD, published by us previously [2] (GEO accession GSE109315). Briefly, mice were sacrificed 6-8 days after the last CDSD session and RNA was extracted with TriReagent (Ambion). Sequencing libraries were prepared with ScriptSeq v2 RNA-seq library preparation kit (Epicentre) and sequencing was performed on NextSeq500 (single-end 96 bp; Illumina). Differential expression analysis on voom normalized [3] gene expression values were performed using limma eBayes [4,5], comparing resilient and susceptible mice to their same-strain controls. Here, we conducted gene set enrichment analysis (GSEA Desktop v4.1.0 [6,7]) using the differential expression results published in [2]. For the GSEA, we ranked the differential expression gene lists by interaction of p-value and fold change of the genes (logFC*p-value). We then analyzed these lists for enrichment of genes belonging to Gene Ontology (GO) terms of node of Ranvier (GO:all, N=40), node (GO:0033268, N=15), paranode (GO:0033270, N=11), juxtaparanodes (GO:0044224, N=10) and internode (GO:0033269, N=4).

Experiment 2: DREADD - Stereotactic viral injections

Mice were anesthetized using 5% isoflurane. Once anesthetized (toe-pinch and tail-pinch reflexes were absent) the mouse was transferred onto a stereotactic frame (Kopf) and maintained on 2% isoflurane anesthesia. The injection coordinates for the

mPFC were AP: + 2.22 mm, ML: ± 0.35 mm, DV: -2.1 mm, and for the vHPC AP: -3.4 mm, ML: ±2.9 mm, DV: -4.5 mm. AAV_{retro}-hSyn1-chl-EGFP_2A_iCre-WRE-SV40p(A) (AAV_{retro}-Cre) was injected into the mPFC for retrograde transport to vHPC neurons projecting to the mPFC to the injection region. For the vHPC viral construct, mice were randomly assigned to receive either the control virus (AAV8-hSyn1-dlox-mCherry(rev)-dlox-WPRE-hGHp(A) or the DREADD (AAV8-hSyn1-dlox-hM3D(Gq)_mCherry(rev)-dlox-WPRE-hGHp(A). All viral constructs were ordered from the Viral Vector Facility at the University of Zürich and ETH Zürich. For each injection, 0.5 µL of the virus was injected using an automated pump (World Precision Instruments) and a 10 µL microsyringe (Hamilton Co.) over the course of 3 minutes. The needle was left in place for another 3 minutes, and then slowly withdrawn (min. 3 minutes). The order of control and DREADD surgeries were balanced across and within days. For post-surgical analgesia, mice were given 5 mg/kg of carprofen subcutaneously before removing from anesthesia. Following surgery, mice were single housed and permitted to recover for 3-4 weeks before beginning behavioral tests, ensuring viral expression.

Experiment 2: DREADD – Behavioral testing and CNO injections

All behavioral tests (except for the SA test) were carried out at the start of the light phase. The mice were brought into the test room 30 minutes before each test. Light conditions for each test are detailed below, and a timeline for behavioral experiments can be seen in Figure 3. Cages were changed once a week, but never within 24h before a behavioral test. For each test the order of mice (control and DREADD) was randomized using random number generation, and the experimenters were blind to the condition of the animal. Mouse movement was recorded and tracked using Ethovision WT (v13, Noldus Technologies).

1. Effects of acute DREADD activation on the elevated zero maze (EZM1)

After acclimatization to the dimly (15 lux) lit room, each mouse received a dose (1 mg/kg) of clozapine-N-oxide (CNO; Abcam, cat. no. ab141704, dissolved in saline) by i.p. injection in their home cage. The elevated zero maze (EZM) test was started 20-30 minutes after this. The mouse was placed into the center of one of two closed sections of a circular maze elevated 40 cm above ground. The total time spent in

open and closed areas was recorded over 5 minutes and analyzed using Ethovision XT10 software. Additionally we defined a 5 cm long zone at the intersection of the open and closed zones as risk assessment zones [8], extending equally (2.5 cm) into the open and closed zones.

CNO injections were continued once per day, between 8am – 10am, for a total of 15 days. The injection order was varied by using one of four randomly generated order lists each day, and the mice were weighed every second day to ensure correct dosing and monitor wellbeing.

2. Effects of chronic DREADD activation on anxiety-like behavior (OFT, EZM2)

On day 13, prior to receiving CNO, we carried out the open field test (OFT). The light conditions were bright to ensure the anxiogenic nature of the test (290 lux). Each mouse was allowed to explore an arena (50 x 50 cm) for 5 minutes. We defined the center of the arena as 5 cm away from the walls at each point. The time the mice spent in the center vs periphery was computed. After the test, each mouse received an injection of CNO and was returned to their home cage.

On day 14, the EZM was repeated with slight modifications (EZM2). To enhance novelty and reduce habituation-induced lack of motivation to explore, we added fresh bedding material to the open zones (changed between each mouse). The apparatus, environmental conditions, and recorded parameters were the same as in EZM1. After the test, the mice received a CNO injection.

3. Effects of chronic DREADD activation on social behavior (SA)

To test for effects of chronic vHPC-mPFC activation on social avoidance behavior, we performed the SA test at the end of the light phase of day 15. To avoid all acute effects of CNO, the mice did not receive an injection this morning. After acclimating to the test room, each mouse went through two trials of the SA test similarly as after CSDS (see above). Here, a naïve male wild-type B6 mouse was used as a social target. The time spent in the IZ and the social interaction (SI) ratio were computed.

4. Effects of an acute re-activation of the chronically activated projection on anxiety-like behavior (EPM).

To explore whether the chronic activation had affected the acute response to CNO, we performed an additional test of anxiety-like behavior following a priming injection. The morning after the SA test (day 16) each mouse received an injection of CNO in the behavioral test room, and after 20-30 minutes they were tested in an elevated plus maze (EPM). The EPM measures anxiety-like behavior with similar parameters as the EZM, but the novel apparatus was expected to minimize habituation-related lack of exploratory drive. To start the test, mice were placed in the center of the apparatus and allowed to freely explore the two opposing closed arms, and the two opposing open arms. Time spent in each arm type was tracked, along with time spent in the center area (as a proxy for risk assessment behavior) [8].

<u>Experiment 1- Nodes of Ranvier immunohistochemistry</u>

We anesthetized the mice 6-8 days after CSDS with a lethal dose of pentobarbital (Mebunat Vet 60 mg/ml, Orion Pharma). Mice were then transcardially perfused with 37°C 4% paraformaldehyde (PFA) in PBS. After post-fixation in 4% PFA (24 h, +4°C), we cut the brains into 40 µm coronal sections with a Leica VT-1200S vibratome (Leica Biosystems), stored in cryoprotectant as free-floating sections (-20°C) until staining.

For paranode staining in the mPFC, sections were first incubated in 0.5% H_2O_2 in TBS for 10 min in RT. Sections were then mounted and stained overnight in +4°C with a cocktail of rabbit anti-Nav1.6 (1:250, #ASC-009, Alomone labs) and mouse anti-CASPR (1:500, #75-001, Neuromab) in 5% NGS 0.5% TBS-T, for nodal and paranodal region staining, respectively. Secondary antibodies were goat anti-rabbit IgG Alexa Fluor 568 (1:400, #A-11011, ThermoFisher Scientific) and goat anti-mouse IgG Alexa Fluor 488 (1:400, #A28175, ThermoFisher Scientific) in 1% NGS in 0.5 % TBS-T. After the last wash, slides were coverslipped with Vectashield + DAPI mounting medium (#H-1200, Vector Laboratories). Co-staining with paranode and juxtaparanode markers was done as follows. Free floating mPFC sections were rinsed 3 times for 10 min in PBS, followed by blocking (5% NGS, 2.5% BSA, 0.25% Triton X-100) for 1 h in RT and incubation with primary antibodies mouse anti-CASPR (1:500, #75-001, Neuromab) and rabbit anti-Kv1.1 (1:300, #APC-009,

Alomone labs) in blocking solution overnight in +4°C. Sections were then rinsed 4 times for 10 min in PBS, followed by secondary antibody incubation with goat anti-mouse Alexa Fluor 555 (1:400, #A-21422, ThermoFisher Scientific) and goat anti-rabbit Alexa 488 (1:400, #A-21422, 1:400, #A28175, ThermoFisher Scientific) in blocking solution for 2 h in RT. After the incubation, sections were rinsed 4 times for 10 min in PBS, then mounted and coverslipped with Vectashield + DAPI mounting medium (#H-1200, Vector Laboratories).

Experiment 2 – Verification of virus injection-sites

The day after the last behavioral test we anesthetized the mice with a lethal dose of pentobarbital (Mebunat Vet 60 mg/ml) and transcardially perfused them with ice cold PBS followed by ice cold 4% PFA in PBS, followed by 24 h post-fixation in +4°C. Sagittal sections were cut using a cryostat (Leica RM2235 microtome, Leica Biosystems) at 35 µm. Serial sections were washed with PBS and mounted with ProLong Diamond hardset mounting medium. The innate fluorescence of the eGFP and mCherry were detected using 3DHISTECH Pannoramic 250 FLASH II digital slide scanner. Mice with bilateral expression of eGFP in the mPFC and mCherry expression in the ventral hippocampus (including CA1/3 subregions) were included in the analysis.

Experiment 2 – Nodes of Ranvier immunohistochemistry

Free-floating sagittal sections were rinsed 3 times for 10 minutes in PBS, followed by incubation for 1h at RT in blocking solution (5% NGS, 2.5% BSA, 0.25% Triton X-100 in PBS). After blocking, the sections were incubated overnight in +4°C with anti-mouse CASPR (1:500, #75-001, Neuromab) in blocking solution. This was followed by rinsing 4 times for 10 min in PBS, and incubation with goat anti-mouse Alexa 647 (1:400, #ab150115, Abcam) in blocking solution for 2h at RT. Thereafter, sections were mounted and coverslipped with mounting medium (Immuno-mount, Thermoscientific).

Imaging

Experiment 1: Imaging was performed with ZEISS LSM 880 Confocal Laser Scanning microscope with AiryScan (Zeiss). The distance from the bregma and the position of the ACC (layer 5/6) or forceps minor for each section was first determined at 10X magnification with a mouse brain atlas[9]. Nodes were then identified with a 63X oil objective in layers V/VI of the ACC and in the forceps minor. To image individual nodes within a field of view, a region around a node was cropped, and a z-stack of the cropped region was acquired. Z-stacks were acquired at a resolution of 0.04 x 0.04 x 0.10 µm.

Experiment 2: Imaging was performed as above but the hippocampal fimbria was first identified using 20X magnification. Paranodes, identified with 63X oil objective, overlapping with mCherry axons (mCherry+) as well as paranodes that did not co-localize with mCherry (mCherry-) were imaged within the hippocampal fimbria.

3D segmentation and morphometry of paranodes and juxtaparanodes

We developed an automated pipeline to segment and analyze the morphology of paranodes and juxtaparanodes, as well as to measure the length of nodes of Ranvier in the acquired 3D microscopy images. The pipeline initially segmented paranodes and juxtaparanodes applying geometric deformable models. However, because more than one pair of paranodes or juxtaparanodes were captured in the acquired images, the pipeline determined the main orientation of the segmented paranodes and juxtaparanodes and excluded those not along the main orientation, as shown in **Figure S4a-f**.

In more detail, we first applied a 3D median filter using a 5 x 5 x 3 sliding window to denoise the acquired 3D images of paranodes (red channel) and juxtaparanodes (green channel) separately to each channel. For segmentation we fused the median-filtered red and green channels into a single channel 3D image, denoted as I, by taking the maximum intensity value between the two channels at each voxel (**Figure S4a**). To segment the 3D image I, first, we applied Frangi filtering[10] to I to enhance its curvilinear structures, i.e., paranodes and juxtaparanodes, and suppress the

background. Then, we thresholded the enhanced image to generate a 3D binary image used to initialize the Chan-Vese active surface model [11]. We used the implementation of the Chan-Vese model available in Matlab's Image Processing Toolbox (version 2018b). We set the parameters as follows: contraction bias was 0.1, smoothness factor was 0.1, and the maximum number of iterations was 100. Applying the connected component analysis to the segmentation result, we generated a preliminary segmentation of paranodes and juxtaparanodes denoted as L (**Figure S4b**). To exclude segmented components other than the paranodes and juxtaparanodes of interest, we first generated a 2D maximum intensity projection of the label image L along the direction of the focal plane, z-axis, as shown in **Figure S4c**. We applied Hough transform [12] to the maximum projection image of L to detect line segments in the image. We used the slope of the longest detected line segment, which represented the main orientation of the segmented paranodes and juxtaparanodes, to draw a line l^* that expanded to the image borders. The dashed line in Figure S4c shows l^* in the maximum projection image of L associated with the main orientation of the segmented components. Then each segmented component was projected on l^*, and its projection length was measured (**Figure S4d**). The segmented components associated with the two longest projections, with non-intersecting projections, were selected as the final labels for the paranodes and juxtaparanodes of interest. For that, we first selected the longest projection and then the second longest projection that did not intersect with the longest projection. Because we applied the segmentation on the fused image, we used the two final segmented components as the initialization surfaces to segment paranodes and juxtaparanodes on 3D median-filtered images separately, using the Chan-Vese model with the same parameter settings as described earlier. **Figures S4e** and **f** show the segmentation boundary of the paranode and juxtaparanode of interest in their corresponding channels.

We quantified morphological aspects of the segmented paranodes and juxtaparanodes in 3D following the approach in references[13,14]. We first extracted the skeleton of paranodes by applying a distance transform-based skeletonization method from[15] (**Figure S4g_1 and g_2**). With a plane perpendicular to the skeleton, we automatically extracted cross-sections along the length of segmented paranodes.

The cross-sectional morphology of paranodes was quantified by the equivalent diameter and the length of the minor and major axes of the fitted ellipse. Moreover, we measured the length of paranodes by measuring the arc length of the acquired skeletons, as shown in **Figure S4g**. The same procedures were applied to analyze the morphology of juxtaparanodes. Denote the set of voxel coordinates in two distinct paranodes by A and B. We measured the length of a node of Ranvier, **Figure S4g2**, by using a robust version of $D(A,B) = \min_{b \in B} \min_{a \in A} d(a,b)$, where $d(.)$ is the Euclidean distance between two points. Define the distance between the set of points S and a point r as $d(r,S) = \min_{s \in S} d(r,s)$. Then, the robust distance between two paranodes was defined as follows:

$$D_{robust}(A,B) = \max(\mathrm{P}_2[\{\mathrm{d(a,B)}, \mathrm{a} \in \mathrm{A}\}], \mathrm{P}_2[\{\mathrm{d(b,A)}, \mathrm{b} \in \mathrm{B}\}]) \qquad (1)$$

where P_2 is the 2nd percentile.

<u>Statistical analysis</u>

We assessed group differences in node and paranode morphology using a mixed model design, in which individual mice and paranode dependency (two paranodes originate from the same node are presumed to be non-independent) were treated as random factors and group (control, resilient and susceptible) and staining batch as fixed factors. We compared behavioral test differences between groups using an unpaired (two-tailed) Student's t test or a Mann-Whitney U test in case data were non-normally distributed. Two-way repeated ANOVA was used to analyze repeated testing in the EZM task. Mixed model analysis was performed using R (4.2.2) and other statistical analyses using Prism 8.

Supplementary Figures 1-4

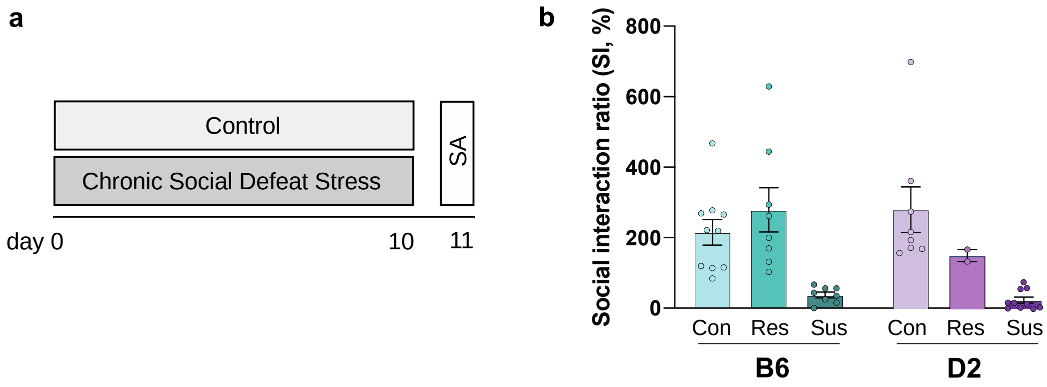

Supplementary Figure 1. Grouping of mice to stress-susceptible and -resilient based on social interaction ratio. a Experimental timeline. **b** Grouping of mice into stress-resilient and -susceptible individuals, similar as in[2]. SA=Social approach test. SI ratio=time spent in interaction zone when a social target present / time spent in interaction zone when no social target present. B6: C57BL/6NCrl; D2: DBA/2NCrl; Con: control; Res: resilient; Sus: Susceptible. Error bars represent ±SEM.

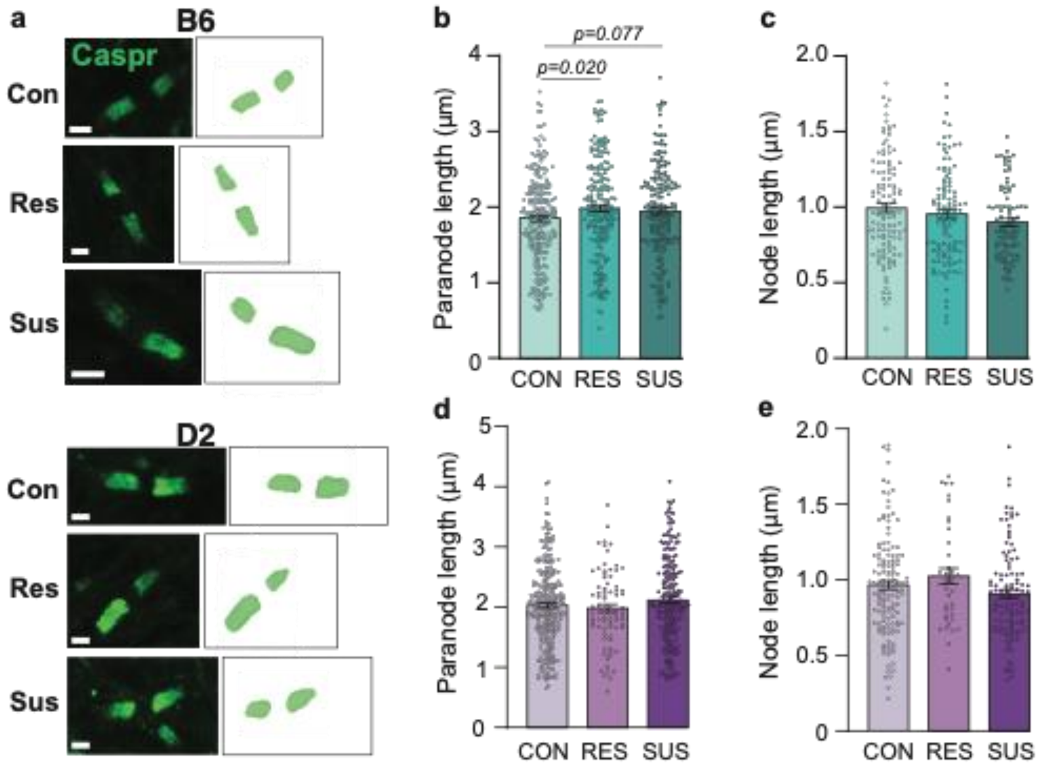

Supplementary Figure 2. Shorter white matter node gaps in stress-susceptible D2 mice.

a 3D reconstruction of paranodes in the forceps minor. **b-e** Quantification of paranode length (**b,d**) and node width (**c,e**) in the forceps minor white matter. (**b** Statistical differences were identified by linear mixed-effect modeling with pairwise comparisons. Paranode length B6: Con *vs*. Res, $t(13.5)=2.640$, $p=0.020$; Con *vs*. Sus, $t(11.5)=1.393$, $p=0.077$. **c** Node length Con *vs*. Res, $t(14.3)=-0.540$, $p=0.597$; Con *vs*. Sus, $t(14.0)=-0.981$, $p=0.343$. **d** Paranode length D2: Con *vs*. Res, $t(9.6)=-0.311$, $p=0.762$; Con *vs*. Sus, $t(7.2)=0.595$, $p=0.570$. Error bars represent ±SEM. B6: C57BL/6NCrl; D2: DBA/2NCrl; Con: control; Res: resilient; Sus: Susceptible.

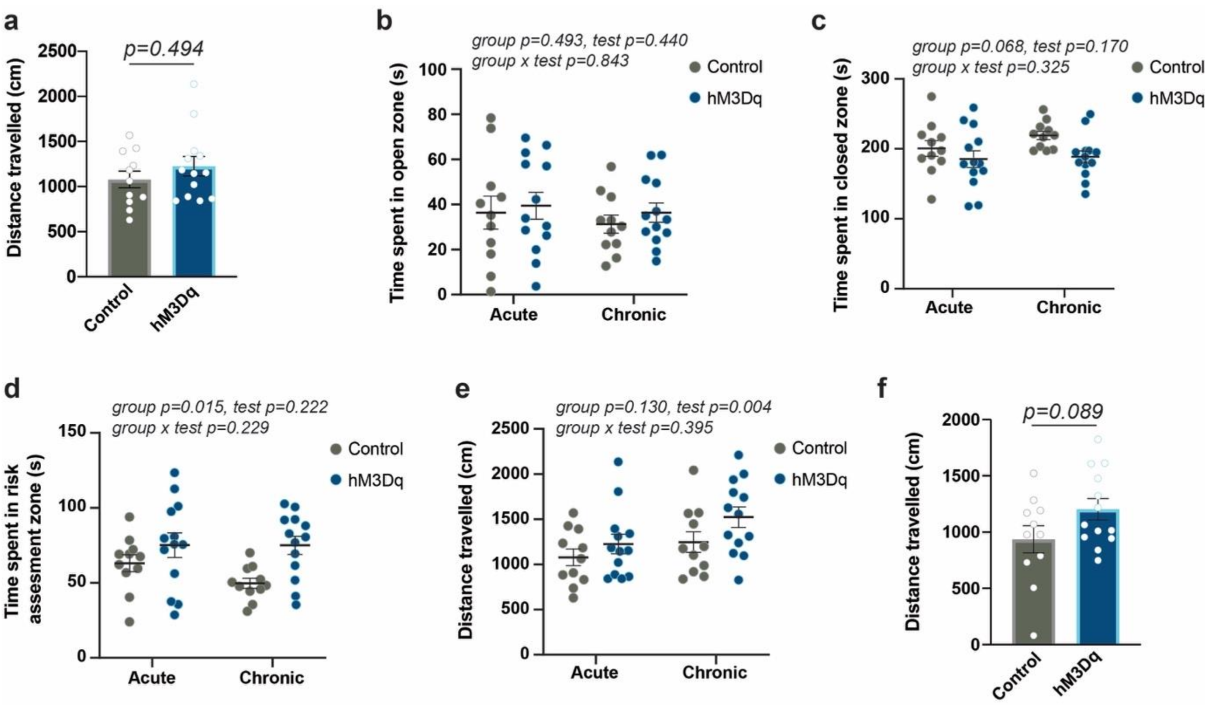

Supplementary Figure 3. Behavior in the EZM and EPM tests after an acute and chronic manipulation of vHPC-to-mPFC projection.

a No differences in locomotor activity after an acute CNO injection in the elevated zero maze (EZM) test (two-sided student's t-test). **b-e** Comparison of time spent in the open (**b**), closed (**c**), risk assessment (**d**) zones and distance travelled (**e**) in the EZM tests after an acute (EZM1) and chronic (EZM2) CNO administration (Two-way

repeated ANOVA). **f** No differences in locomotor activity after chronic CNO injections in the elevated plus maze (EPM) test (two-sided student's t-test). Control n=11; hM3Dq n=13. Error bars represent ±SEM.

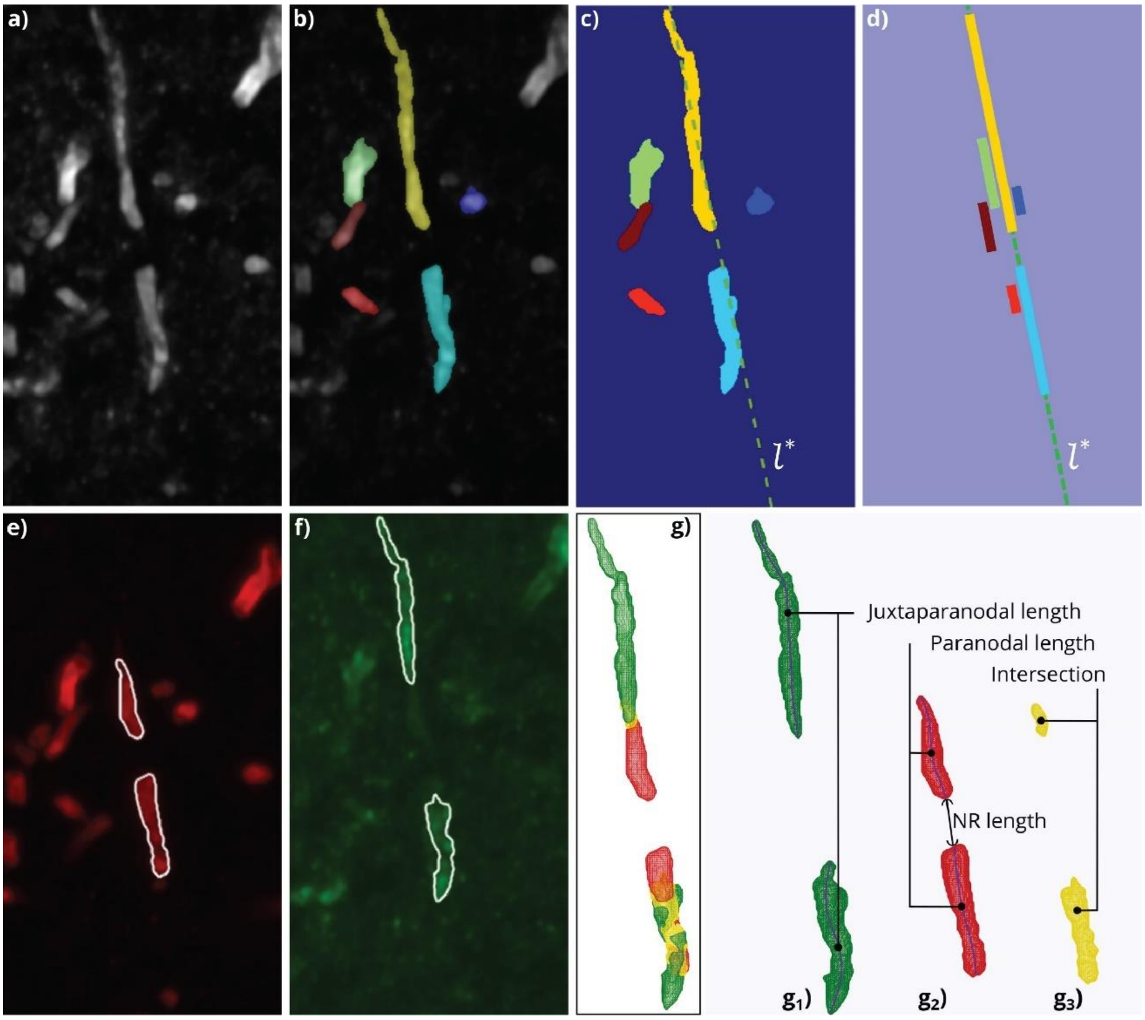

.

Supplementary Figure 4. Automated segmentation and 3D morphometry of paranodes and juxtaparanodes in confocal microscopy images. **a** A maximum projection image of paranodes (red channel) and juxtaparanodes (green channel). **b** A preliminary segmentation of paranodes and juxtaparanodes using the Chan-Vese active surface model. **c** The slope of the longest detected line segment, which associates with the main orientation of the segmented components, was used to draw a line *l** (dashed line) that expanded to the image borders. **d** Projection of the segmented components on *l**. Colors correspond with the segmented components in panel c. **e** Segmentation of paranodes visualized on the maximum projection image of the red channel. **f** Segmentation of juxtaparanodes visualized on the maximum projection image of the green channel. **g** 3D rendering of juxtaparanodes (green, g_1,

paranodes (red, g_2, and their intersection (yellow, g_3). The extracted skeletons of the paranodes and juxtaparanodes are overlaid on their corresponding rendering. NR=node of Ranvier.

References

1. Golden, S. A., III, H. E. C., Berton, O. & Russo, S. J. A standardized protocol for repeated social defeat stress in mice. *Nature Protocols* **6**, 1183 (2011).
2. Laine, M. A. *et al.* Genetic Control of Myelin Plasticity after Chronic Psychosocial Stress. *eNeuro* **5**, ENEURO.0166-18.2018 (2018).
3. Law, C. W., Chen, Y., Shi, W. & Smyth, G. K. voom: precision weights unlock linear model analysis tools for RNA-seq read counts. *Genome Biol* **15**, R29 (2014).
4. Ritchie, M. E. *et al.* limma powers differential expression analyses for RNA-sequencing and microarray studies. *Nucleic Acids Research* **43**, e47–e47 (2015).
5. Phipson, B., Lee, S., Majewski, I. J., Alexander, W. S. & Smyth, G. K. Robust hyperparameter estimation protects against hypervariable genes and improves power to detect differential expression. *Ann. Appl. Stat.* **10**, (2016).
6. Subramanian, A. *et al.* Gene set enrichment analysis: A knowledge-based approach for interpreting genome-wide expression profiles. *Proceedings of the National Academy of Sciences* **102**, 15545–15550 (2005).
7. Mootha, V. K. *et al.* PGC-1α-responsive genes involved in oxidative phosphorylation are coordinately downregulated in human diabetes. *Nat Genet* **34**, 267–273 (2003).
8. Lebow, M. *et al.* Susceptibility to PTSD-Like Behavior Is Mediated by Corticotropin-Releasing Factor Receptor Type 2 Levels in the Bed Nucleus of the Stria Terminalis. *Journal of Neuroscience* **32**, 6906–6916 (2012).
9. Franklin, K. B. J. *The mouse brain in stereotaxic coordinates*. (Elsevier Academic Press, 2008).

10. Frangi, A. F., Niessen, W. J., Vincken, K. L. & Viergever, M. A. Multiscale vessel enhancement filtering. in *Medical Image Computing and Computer-Assisted Intervention — MICCAI'98* (eds. Wells, W. M., Colchester, A. & Delp, S.) vol. 1496 130–137 (Springer Berlin Heidelberg, 1998).
11. Chan, T. F. & Vese, L. A. Active contours without edges. *IEEE Trans. on Image Process.* **10**, 266–277 (2001).
12. Duda, R. O. & Hart, P. E. Use of the Hough transformation to detect lines and curves in pictures. *Commun. ACM* **15**, 11–15 (1972).
13. Abdollahzadeh, A., Belevich, I., Jokitalo, E., Tohka, J. & Sierra, A. Automated 3D Axonal Morphometry of White Matter. *Sci Rep* **9**, 6084 (2019).
14. Abdollahzadeh, A., Belevich, I., Jokitalo, E., Sierra, A. & Tohka, J. DeepACSON automated segmentation of white matter in 3D electron microscopy. *Commun Biol* **4**, 179 (2021).
15. Abdollahzadeh, A., Sierra, A. & Tohka, J. Cylindrical Shape Decomposition for 3D Segmentation of Tubular Objects. *IEEE Access* **9**, 23979–23995 (2021).
16. Hanley, J. A. Statistical Analysis of Correlated Data Using Generalized Estimating Equations: An Orientation. *American Journal of Epidemiology* **157**, 364–375 (2003).
